# Effects of Four Weeks of Alternate-Day Fasting with or Without Protein Supplementation—A Randomized Controlled Trial

**DOI:** 10.3390/nu17233691

**Published:** 2025-11-25

**Authors:** Benedict Wei Jun Pang, Yifan Yang, Nur Rashiqah, Christopher Bingqiang Huang, Da Wei Sim

**Affiliations:** 1 Physical Education and Sports Science, National Institute of Education, Nanyang Technological University, Singapore 637616, Singapore; benedict.wj.pang@gmail.com (B.W.J.P.);; 2 Geriatric Education and Research Institute, Singapore 768024, Singapore; 3 Science of Learning in Education Centre, National Institute of Education, Nanyang Technological University, Singapore 637616, Singapore

**Keywords:** weight loss, muscle preservation, intermittent fasting, alternate-day fasting, short-term, body composition, blood glucose, blood pressure

## Abstract

**Background/Objectives**: Long-term alternate-day fasting (ADF) effectively combats obesity, but its short-term effects are less clear. Like other diets, ADF-induced weight loss often includes muscle loss, and whether protein supplementation mitigates this is uncertain. This study examined the effects of short-term ADF on body composition and health and whether protein supplementation preserves muscle mass during weight loss in young Asian men with an unhealthy BMI (≥23.0 kg/m^2^). **Methods**: Twenty participants were recruited for a single-arm trial to address the first objective, and twenty-six participants were randomly assigned to a control (C) or protein group (P) in a follow-up trial to address the second objective. The participants alternated between feeding (ad libitum) and fasting (400–600 kcal consumed between 12 and 2 PM) days for four weeks. The participants in P consumed 25 g of whey protein as part of the fasting-day meal. Pre–post body composition was assessed using bioelectrical impedance analysis. Anthropometry, fasting blood glucose (FG), and resting blood pressure (BP) were measured weekly. **Results**: Since interaction effects were absent, data from all three groups were combined for analyses. Four weeks of ADF significantly (*p* < 0.001) reduced body (2.4 kg), fat (1.6 kg), and fat-free (0.8 kg) mass. BP and FG levels remained unchanged (*p* = 0.753–0.919). No significant differences were detected between the C and P groups for any of the measures. **Conclusions**: Short-term ADF effectively reduced body and fat mass, but it also reduced muscle mass, and this reduction was not attenuated by low-dose protein supplementation (25 g) during fasting days. Future studies should explore the effectiveness of protein or leucine supplementation, throughout the feeding and fasting days, in terms of preserving muscle during weight loss.

## 1. Introduction

Globally, 43.5% of adults (age-standardized) are overweight (BMI: 25.0–29.9 kg/m^2^) and are 15.8% obese (≥30.0 kg/m^2^) [1], making obesity a global health epidemic. In Singapore, 40.7% and 13.9% of adults are overweight and obese, respectively, with higher prevalence when guidelines for increased cardiovascular disease (CVD) risk in Asians are used instead (23.0–27.4 kg/m^2^ and ≥27.5 kg/m^2^) [2,3]. Given that obesity is a risk factor for chronic diseases, including metabolic syndrome, CVD, diabetes, and cancer, and overall mortality [4], its increasing prevalence constitutes a growing public health concern.

To combat obesity, various weight loss strategies have been promoted. Recently, the use of intermittent fasting (IF) has gained a significant amount of interest due to its purported benefits regarding lipid profiles [5] and cardiovascular and metabolic health [6], in addition to promoting weight loss [7]. Essentially, IF involves alternating between periods of food abstinence (fasting) and food consumption (feeding) [8]. Fasting periods can range from 16 to 20 h/day to up to 48 h/week [7]. In particular, alternate-day fasting (ADF), which involves alternating between feeding (ad libitum food intake) and fasting days (a small meal of ~400–600 kcal or 25% of daily caloric needs) every 24 h, has been one of the most well-researched and popularized IF protocols [9].

The health benefits associated with ADF are manifold. Current evidence suggests that ADF reduces BMI, body mass, fat mass, total and low-density lipoprotein cholesterol, triglyceride levels, and systolic and diastolic blood pressure [10,11]. Compared with very-low-calorie dieting (VLCD), 3–12 weeks of ADF leads to a greater reduction in fat mass and a lower reduction in fat-free mass while decreasing hunger and improving satiety and dieting satisfaction, illustrating superior adherence rates and ease of compliance [12]. Of note, a longer-term study lasting six months showed non-superior adherence rates with ADF compared to a traditional calorie restriction diet [13]. Regardless, the period for existing ADF studies typically ranges from 8 to 48 weeks, long durations that are hardly motivating to individuals looking to lose weight [10,11,12]. Moreover, short-term weight cuts while maintaining muscle mass would be relevant to athletes, especially those participating in sports with weight categories or in which aesthetics play a significant role. To date, only a handful of studies have investigated the effects of short-term (2–4 weeks) ADF, all of which were conducted on predominantly Caucasian populations [14,15,16,17,18,19,20]. Collectively, these studies showed that two weeks of ADF was insufficient to induce weight loss in overweight young men [16], and at least 3–4 weeks was required to reduce body mass and fat mass in non-obese and obese young to middle-age adults [14,15,17,18,19,20]. It remains unclear whether short-term ADF can improve body composition in a mixed normal-weight-to-obese group, especially among Asian populations with greater CVD risks (BMI ≥ 23.0 kg/m^2^).

Despite the purported health benefits associated with ADF, as with other weight loss diets, muscle loss is a major concern [21]. Importantly, studies comparing ADF with no-diet controls consistently report significant muscle loss in the ADF group [11]. Additionally, while some studies suggest that high protein intake may preserve muscle during weight loss [22,23], others found no differences between high-protein groups and controls regarding muscle preservation [24,25]. At present, there is no evidence suggesting protein has preservatory effects on muscle during ADF.

Considering these gaps, our objectives for this study are to investigate (1) whether short-term (4-week) ADF has beneficial effects on body composition and (2) if increased protein intake through whey protein supplementation can preserve muscle during weight loss among Asian young men with unhealthy BMI levels. To achieve these objectives, a single-arm pilot study was conducted with the main aim of addressing the first objective, and a follow-up randomized-controlled trial (RCT) was conducted to address the second objective. Additionally, the effects of 4-week ADF on other health markers, including fasting blood glucose and resting blood pressure, were explored. We hypothesized that 4-week ADF is sufficient to induce weight loss, and that protein supplementation during fasting days can reduce diet-induced muscle loss in normo-weight to obese young men with increased cardiovascular risks (BMI ≥ 23.0 kg/m^2^).

## 2. Materials and Methods

### 2.1. Study 1 Design

In this single-arm study (S), 20 young men (21–35 years) with increased CVD risks (BMI ≥ 23.0 kg/m^2^) were recruited and subjected to four weeks (28 days) of ADF. Studies that found effective weight loss following ADF used a sample size of 8 or 16 [15,17,26]; hence, we aimed for a sample size of 20 (based on changes in total body mass) to buffer for dropouts. Body composition was assessed pre- and post-intervention, while anthropometry, fasting blood glucose, and resting blood pressure were measured weekly. Participants completed a weekly physical activity questionnaire and provided dietary records. Ethics approval was obtained from the Nanyang Technological University (NTU) Institutional Review Board (IRB-2016-09-055). There were no changes to methods or outcome measures after trial commencement.

#### 2.1.1. Participant Screening

Participants were screened according to the following inclusion criteria: (1) male, (2) 21–35 years old, (3) does not smoke or use tobacco products (including shisha), and (4) BMI ≥ 23.0 kg/m^2^. Additionally, participants who met any of the following exclusion criteria were excluded from the study: (1) being unable to adhere to physical activity and diet requirements; (2) having failed an exercise stress test; (3) having a health condition(s) that might be worsened with fasting; (4) taking long-term medication(s) for heart, blood, lung, liver, kidney, or joint condition(s); and (5) taking other long-term alternative medication, including traditional Chinese medicine, that may affect the study’s measurements. Eligible participants signed informed consent before their inclusion in the study.

#### 2.1.2. ADF Protocol

The ADF protocol began at 12 AM and alternated between fasting and feeding days in 24 h cycles. On fasting days, no food and beverages were allowed except for plain water and/or zero-calorie beverages and a small meal corresponding to 400–600 kcal consumed [9] between 12–2 PM to standardize the total duration of fasting and feeding. On feeding days, food and beverages were allowed to be consumed ad libitum. Participants maintained consistent physical activity levels throughout the study.

#### 2.1.3. Laboratory Sessions

Participants visited the laboratory (Human Bioenergetics Laboratory, Physical Education and Sports Science, National Institute of Education, NTU, Singapore) on 5 occasions across 5 weeks. They abstained from engaging in intentional physical activity (except activities of daily living) for at least 48 h and drinking alcohol and/or caffeine for at least 24 h, and they fasted overnight for at least 10 h before each laboratory session.

#### 2.1.4. Body Composition

Body composition was assessed pre- and post-intervention (first and fifth laboratory sessions) through bioelectrical impedance analysis (BIA) using the InBody 720 (Biospace Co., Ltd., Seoul, Republic of Korea), wherein measurements of total body fat mass (FM), fat-free mass (FFM), skeletal muscle mass (SMM), appendicular fat-free mass (AFFM), percentage of body fat (PBF), visceral fat area (VFA), and hydration status (intracellular water/extracellular water (ICW/ECW)) were obtained. FM index (FMI), FFM index (FFMI), SMM index (SMMI), and AFFM index (AFFMI) were calculated using the respective measures in kilograms divided by square of height in meters (kg/m^2^). The use of multi-frequency BIA for the purpose of assessing body composition has been extensively validated in comparison to gold-standard methods [27,28,29] and used in other weight loss studies [17,26,30]. Participants started their 4-week ADF intervention within 1 week from the first session.

#### 2.1.5. Anthropometry

Body height (m) and mass (kg) were measured weekly using the ID1Plus electronic weighing scale (Mettler Toledo, Singapore) and seca 242 stadiometer (seca GmbH & Co., KG, Hamburg, Germany), respectively. BMI was calculated using body mass in kilograms divided by the square of height in meters (kg/m^2^).

#### 2.1.6. Health Assessments

Fasting blood glucose (FG) and resting blood pressure (BP) were also measured weekly. For FG (mmol/L), a tiny drop of blood was obtained through finger-pricking using an ACCU-CHEK Safe-T-Pro Plus lancet (Roche Diabetes Care GmbH, Mannheim, Germany) and analyzed using a OneTouch Ultra 2 blood glucose meter (LifeScan, Malvern, PA, USA). Resting systolic (SBP) and diastolic (DBP) blood pressure (mmHg) were assessed using an OMRON HEM-907 blood pressure monitor (OMRON healthcare Co., Ltd., Kyoto, Japan).

#### 2.1.7. Physical Activity Questionnaire and Dietary Record

The global physical activity questionnaire (GPAQ) [31] was administered weekly to estimate physical activity levels (MET·min), though it should be noted that GPAQ does not capture exercise type. Participants completed a 2-day dietary record of any 2 fasting days each week, and total energy intake (kcal), protein (g), fat (g), and carbohydrate (g) content were analyzed for each recorded day.

### 2.2. Study 2 Design

**Figure 1:**
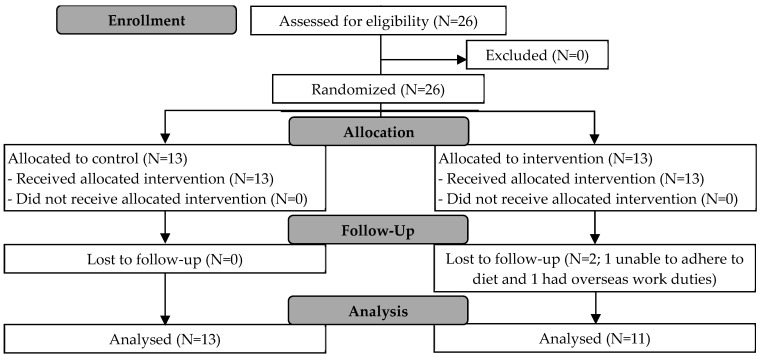
CONSORT participant flow diagram for Study 2.

In this two-arm RCT, 26 young men (21–35 years) with increased CVD risks (BMI ≥ 23.0 kg/m^2^) were recruited to undergo four weeks (28 days) of ADF and randomly assigned to one of two groups, namely, a control (C) or protein group (P), in blocks of 2 (CP or PC) to ensure there was an equal sample size in each group. Participants in P additionally consumed protein supplements on the fasting day (Figure 1). The compromise power calculation obtained using G*Power version 3.1.9.2 for a 2 (group) × 2 (time) repeated-measures ANOVA (rANOVA) interaction effect is 0.85 for 26 total participants (based on the final number of 13 participants in Study 1), with an f effect size of 0.25, a beta/alpha ratio of 1, and a correlation of 0.5 among repeated measures. Ethical approval was obtained from the NTU IRB (IRB-2017-08-010). Inclusion/exclusion criteria were identical to those in Study 1, and eligible participants signed informed consent before their inclusion into the study. There were no changes to the methods or outcome measures after trial commencement (ClinicalTrials.gov ID: NCT07241689).

#### ADF Protocol

The ADF protocol was identical to the one used in Study 1, wherein a small, self-selected meal (400–600 kcal) was consumed between 12 and 2 PM on fasting days. For group P, a 25 g whey protein supplement (125 kcal) was consumed as part of the fasting-day meal, with the remaining 275–475 kcal coming from self-selected food items. On feeding days, food and beverages were allowed to be consumed ad libitum.

Apart from the distinction in fasting-day meal for group P, the study design and protocols were identical to those in Study 1, including laboratory measurements, health assessments, and physical activity questionnaires and dietary records.

### 2.3. Statistical Analyses

SPSS Statistics version 27.0 (IBM, Chicago, IL, USA) and JASP version 0.16.3 (JASP Team) were used for data analyses. Differences in body composition pre- and post-intervention (and between groups for Study 2) were first analyzed separately for Study 1 (within-subjects analyses (group S)) and Study 2 [time, group (C and P), and interaction effects]. When no interaction effects were present, data from all three groups were combined for within-subjects analyses. Significance level was set at *p* ≤ 0.05.

For Study 1 (S), within-subjects *t*-tests were conducted for variables with parametric data: BM, FM, FFM, SMM, AFFM, BMI, PBF, VFA, and hydration status (ICW/ECW). Wilcoxon test was performed for non-parametric variables: FMI, FFMI, SMMI, and AFFMI.

For Study 2 (C and P), 2 (groups) × 2 (time: pre–post) rANOVA was conducted for ICW/ECW. For the other variables (BM, FM, FFM, SMM, AFFM, FFMI, SMMI, AFFMI, BMI, PBF, FMI, and VFA), due to non-normality of data, generalized estimating equations (GEE) were employed using a robust estimator, a normal distribution with a log link function, and an exchangeable correlation matrix. Models were first selected based on the smallest goodness of fit, followed by smaller covariances of parameter estimates. There were no significant interactions, and main effects were reported based on estimated marginal means to adjust for unequal sample sizes (if any).

For combined analyses, within-subjects *t*-tests were conducted for variables with parametric data: BM, FM, FFM, SMM, AFFM, BMI, PBF, FMI, VFA, and ICW/ECW. Wilcoxon test was performed for non-parametric variables: FFMI, SMMI, and AFFMI.

Differences in average calories and protein content of the fasting-day meals between Groups P and C were analyzed using between-subjects *t*-test and Welch test, respectively. For all *t*-tests and Welch tests, effect sizes were calculated using Cohen’s *d* (0.2, small; 0.5, medium; 0.8, large). For Wilcoxon tests, effect sizes were calculated using the matched rank biserial correlation coefficient, r_rb_ (0.1, small; 0.3, medium; 0.5, large). For rANOVA, partial eta squared (*η^*2*^p*) effect sizes were used (0.01, small; 0.06, medium; 0.14, large).

As for weekly measurements (i.e., GPAQ, SBP, DBP, and FG), due to non-normality of data, GEE was performed for GPAQ, SBP, and DBP using a robust estimator with a normal distribution with a log link function, along with an autoregressive correlation matrix, while an unstructured correlation matrix was used for FG. Similarly, models were first selected based on the smallest goodness of fit and then smaller covariances of parameter estimates. There were no significant interactions for Study 2, and any significant time effect was followed using simple contrast against the baseline (pre-intervention) with Sidak correction for multiple comparisons. Results were based on estimated marginal means.

## 3. Results

### 3.1. Participants

Among the 20 participants recruited for Study 1 (between February and March 2017) and the 26 participants recruited for Study 2 (between January and March 2018), N = 7 and N = 2 of them dropped out, respectively (8 were unable to commit; 1 was uncontactable). Therefore, data from N = 37 participants (N = 13 and N = 24, respectively) were used for analyses, with a total of N = 13 in Group S, N = 13 in Group C, and N = 11 in Group P. Participant characteristics are displayed in Table 1.

### 3.2. Fasting-Day Meal

**Figure 2:**
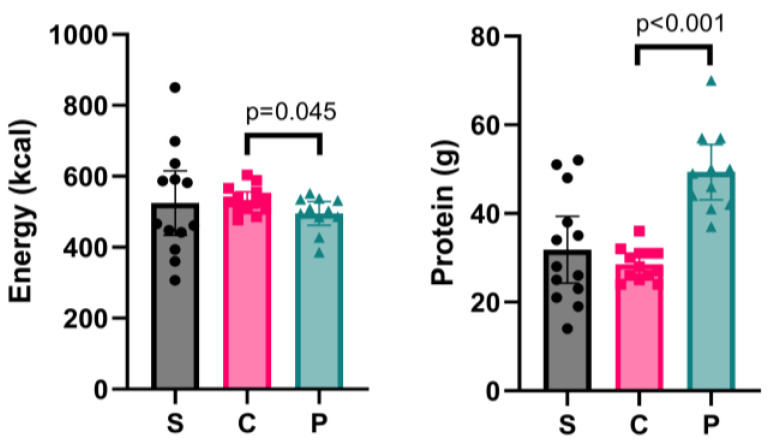
Fasting-meal energy (kcal) and protein (g) intake for Study 1—single-arm group (S) and Study 2—control (C) and protein (P) groups. Bar chart presents mean and 95% CI, with scatterplot of individual data points. Independent samples *t*-test was conducted only for Study 2 to compare the differences between groups C and P.

On fasting days, group S consumed on average 525 (SD 151) kcal for the fasting meal, of which 25 (7)%, 37 (15)%, and 38 (12)% came from protein, fat, and carbohydrates, respectively. Group P consumed 495 (50) kcal, of which 40 (8)%, 26 (6)%, and 34 (13)% came from protein, fat, and carbohydrates. Group C consumed 533 (38) kcal, of which 22 (4)%, 33 (5)%, and 46 (5)% came from protein, fat, and carbohydrates. An independent-samples *t*-test revealed that, despite consuming fewer calories (*p* = 0.045, *d* = −0.9), group P consumed significantly more protein (*p* < 0.001. *d* = 3.0) on the fasting day than group C (Figure 2), as intended.

### 3.3. Hydration Status

ICW/ECW is an indicator of hydration status, which can affect BIA results. There were no significant differences in pre–post ICW/ECW values for group S (*p* = 0.827, *d* = −0.06), and we did not find any significant interactions (*p* = 0.744, *η^2^p* < 0.01) or main effects of time on ICW/ECW for groups P and C (*p* = 0.978, *η^2^p* < 0.01), suggesting that pre–post hydration status was consistent in both studies.

### 3.4. Body Composition

**Figure 3:**
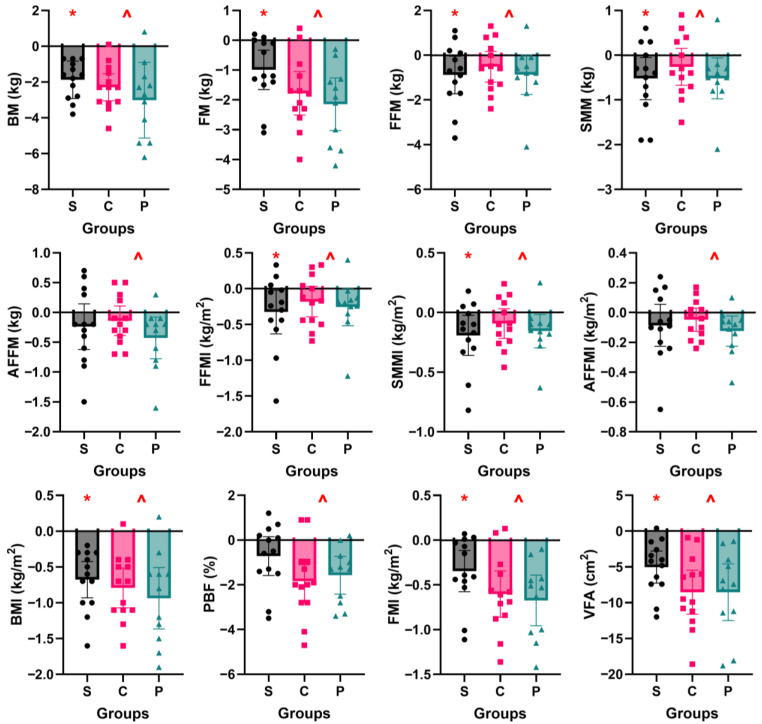
Pre–post changes in body composition for Study 1—single-arm group (S) and Study 2—control (C) and protein (P) groups. Bar chart presents means and 95% CIs, with a scatterplot of individual data points. BM, body mass; FM, fat mass; FFM, fat-free mass; SMM, skeletal muscle mass; AFFM, appendicular fat-free mass; FFMI, fat-free mass index; SMMI, skeletal muscle mass index; AFFMI, appendicular fat-free mass index; BMI, body mass index; PBF, percentage body fat; FMI, fat mass index; and VFA, visceral fat area. Separate statistical analyses were conducted for Studies 1 and 2. * indicates a significant pre–post change for Study 1 (*p* < 0.05). ^ indicates a significant time effect for Study 2 (*p* < 0.05). All interaction effects for Study 2 were not significant (*p* > 0.05).

Post-intervention (Figure 3), group S exhibited significant reductions in BM (*p* < 0.001, *d* = −1.79), FM (*p* = 0.007, *d* = −0.91), FFM (*p* = 0.041, *d* = −0.63), SMM (*p* = 0.032, *d* = −0.67), FFMI (*p* = 0.027, *d* = −0.69), SMMI (*p* = 0.021, *d* = −0.71), BMI (*p* < 0.001, *d* = −1.62), FMI (*p* = 0.006, *d* = −0.82), and VFA (*p* < 0.001, *d* = −1.36). Changes in AFFM (*p* = 0.235, *d* = −0.35), AFFMI (*p* = 0.244, *d* = −0.39), and PBF (*p* = 0.097, *d* = −0.50) were not significant. For groups P and C, there were no significant interaction effects of group and time on any measure (*p* = 0.179–.807). However, a main effect of time was significant for all measures, with a reduction post-intervention: BM (*p* < 0.001, *d* = −0.21), FM (*p* < 0.001, *d* = −0.23), FFM (*p* = 0.004, *d* = −0.11), SMM (*p* = 0.004, *d* = −0.10), AFFM (*p* = 0.003, *d* = −0.08), FFMI (*p* = 0.002, *d* = −0.14), SMMI (*p* = 0.002, *d* = −0.13), AFFMI (*p* = 0.001, *d* = −0.12), BMI (*p* < 0.001, *d* = −0.23), PBF (*p* < 0.001, *d* = −0.26), FMI (*p* < 0.001, *d* = −0.24), and VFA (*p* < 0.001, *d* = −0.27).

**Figure 4:**
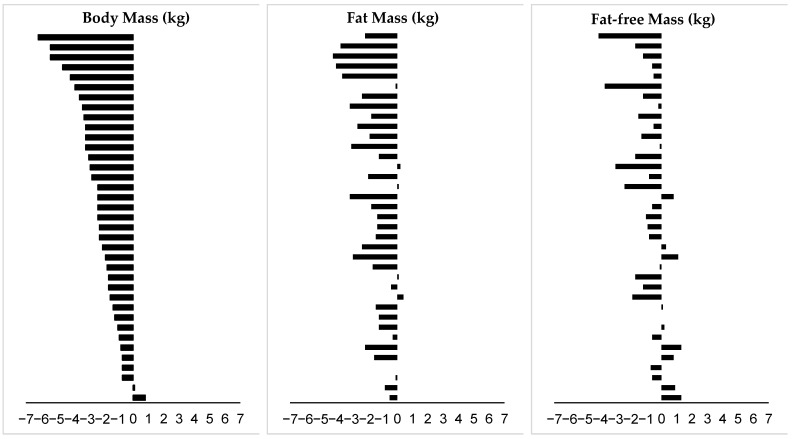
Corresponding changes in body mass, fat mass, and fat-free mass (ranked by change in body mass) for all 37 participants.

Since no significant interaction effects were present between groups P and C, data from all three groups (N = 37) were combined for within-subjects analyses. The results showed significant decreases in all measures of body composition post-intervention, namely, BM, FM, FFM, SMM, AFFM, FFMI, SMMI, AFFMI, BMI, PBF, FMI, and VFA (Table 2). Corresponding changes in BM, FM, and FFM for each participant are depicted in Figure 4 to depict individual responses. All but two participants lost BM (coefficient of variation, CV of change = 0.65), but the changes in FM (CV = 0.78) and FMM (CV = 1.68) were more varied. While the change in BM is correlated with changes in FM (Spearman correlation coefficient, *ρ* = 0.586, *p* < 0.001) and FFM (*ρ* = 0.537, *p* < 0.001), respectively, there is no correlation between FM and FFM changes (*ρ* = −0.291, *p* < 0.081).

### 3.5. Physical Activity and Blood Markers

There were no significant interaction effects between groups P and C in terms of weekly physical activity level [GPAQ (*p* = 0.426)], SBP (*p* = 0.653), or DBP (*p* = 0.818), and thus data from Studies 1 and 2 were combined (Table 3). Analysis of the combined data showed a significant difference in GPAQ levels across time (*p* < 0.001). The simple contrast against baseline revealed significant reductions in GPAQ levels between baseline and the end of week 2 (*p* = 0.002), and between baseline and the end of week 3 (*p* = 0.004).

Analyses of the combined data showed significant differences in DBP (*p* = 0.021) and FG (*p* < 0.001) across time but not for SBP (*p* = 0.562). Post hoc analyses revealed significant reductions in DBP between the baseline and at the end of week 3 (−2.8 mmHg, *p* = 0.028) and in FG between the baseline and the the end of week 1 (−0.22, *p* = 0.026). There were, however, no significant pre–post differences in DBP (*p* = 0.809) or FG (*p* = 0.919) from baseline to post-intervention.

## 4. Discussion

To our knowledge, this is the first study to investigate the effects of short-term ADF on body composition, along with the effects of whey protein supplementation on muscle preservation during short-term ADF, among normal weight to obese (Asian) young men with increased CVD risks.

### 4.1. Effect of Short-Term ADF on Body Composition

Our results showed that four weeks of ADF was sufficient to induce significant reductions (Figure 4) in body mass (35/37 participants) and fat mass (33/37). However, there was a concomitant decrease in muscle (fat-free mass) as well (28/37). Increased protein intake through whey protein supplementation during fasting days did not alleviate muscle loss.

There was a significant reduction in body mass, amounting to 2.4 kg (2.9%), after four weeks of ADF, a rate that is clinically important and within safe recommendations (0.45–0.9 kg/week) [32]. This agrees with the previous literature, where 3–4 weeks [14,15,17,18,19,20], but not 2 weeks [16], of ADF was sufficient and effective for weight loss. Since weight loss is mainly determined by overall energy deficit [33], the energy deficit accumulated with <3 weeks of ADF is likely insufficient to promote significant weight loss, and at least 3–4 weeks is required to accrue a sufficient energy deficit to alter body mass among normal weight to obese individuals. Notably, short-term weight loss can also be attributable to acute losses in water weight, especially during fasting and calorie restriction, due to a depletion in glycogen stores [34]. Each gram of stored glycogen holds 3–4 g of water, which translates to 4–5 g of weight lost for every 1 g of glycogen expended during fasting and calorie restriction [35].

Four weeks of ADF reduced fat mass by an average of 1.6 kg (68.2% of total weight loss), along with other obesity indices, including BMI (−3.0%), FMI (−8.3%), PBF (−5.9%), and VFA (−9.0%), with large effect sizes for all indices. However, this was accompanied by significant muscle loss in terms of FFM (by an average of 0.8 kg, or 31.8% of total weight loss), SMM (−1.2%), AFFM (−1.0%), FFMI (−1.3%), SMMI (−1.3%), and AFFMI (−1.0%), with moderate effect sizes for all indices (Table 2). This is supported by the findings of other weight loss studies, where, regardless of dietary intervention, a significant reduction in lean mass or FFM (25–42% of total weight loss) was expected [12,36]. Due to the physiological demands of calorie restriction and weight loss, there is a natural decrease in basal and postprandial muscle protein synthesis (MPS), which is attributable to the overall lower protein and calorie intake that directly stimulate MPS. These decreases in basal and postprandial MPS, the reduction in the frequency of feeding, and the increase in proteolysis during fasting and calorie restriction [37] collectively exert deleterious effects on muscle regardless of the type of diet implemented [10].

### 4.2. Effectiveness of Protein Supplementation on Fasting Day

Interestingly, whey protein supplementation—despite increasing fasting-day protein intake significantly, raising it by 1.7× in group P (49.4 g) compared to group C (28.5 g)—was not effective in attenuating muscle loss. Corroboratively, several studies have reported that increased protein intake alone during energy restriction and weight loss does not improve or preserve muscle [24,25,38]. One possible reason for the absence of a significant effect could be that the difference in body-mass-adjusted protein consumption between groups P (0.55–0.57 g/kg/day) and C (0.38–0.39 g/kg/day) was too small (0.16–0.19 g/kg/day) to result in any meaningful difference. Notably, fasting-day protein intake for both groups (0.38–0.57 g/kg/day) was below the recommended dietary allowance of 0.8 g/kg (body mass)/day, an amount typically recommended to prevent protein deficiency [39]. Nonetheless, it is hardly feasible to enforce a fasting-day protein intake of 0.8 g/kg/day, as that would represent an average protein intake of ~71.5 g (286 kcal) for the P group, which equates to 47.7–71.5% of total fasting-day calories (400–600 kcal) coming from protein alone. Additionally, protein intake on feeding days was not measured or controlled in these studies. Though consistent with the nature of ADF and its evaluation for effectiveness, the lack of monitoring on feeding days allows for unknown dietary compensation between groups. As such, a further explanation as to why group P did not experience greater muscle preservation could be that the protein intake on feeding days was not sufficient to compensate for the lower amount of protein consumed on fasting days in either group. In fact, the protein intake on feeding days may have been insufficient in both groups, resulting in comparable losses in muscle mass.

Indeed, a previous meta-analysis found that when individuals adhered to a weight loss diet, high protein intakes averaging 1.25 g/kg/day resulted in greater reductions in body mass and fat mass, relative to a standard protein intake of 0.72 g/kg/day, while preserving FFM [23]. Future ADF studies could explore the effect of increasing protein intake on both the feeding and fasting days in order to bring the total daily and/or weekly protein intake, averaged across both feeding and fasting days, closer to the amount of 1.25 g/kg/day or higher.

Importantly, recent studies have demonstrated that protein quality (the amount of leucine) may be a better determinant of MPS and muscle anabolism than protein quantity [40,41]. While leucine intake was not monitored in the present study, there is merit in exploring the effectiveness of leucine interventions on muscle preservation during weight loss in future studies. Given the important role that leucine plays in activating MPS, it is possible that leucine interventions can help to attenuate muscle loss during weight loss.

Nonetheless, increased protein consumption can improve satiety [42], which could explain the small (38 kcal) but significant (*p* = 0.045) lower caloric intake on fasting days in P group compared to C group. Although no significant differences were detected in terms of weight loss between groups, decreased energy intake and improved satiety through increased protein consumption could result in superior weight loss that is clinically relevant over longer periods of time.

### 4.3. Potential Influence of Physical Activity Type on Muscle Mass Preservation

Physical activity and resistance-training habits could have further confounded the effects of protein supplementation on muscle preservation. While our GPAQ analyses revealed no significant changes in physical activity levels and no interaction effect between groups P and C throughout the intervention, physical activity was quantified using energy expenditure (MET·min/week) and not differentiated by the type of activity. Given that resistance training serves as a potent anabolic stimulus for muscle hypertrophy through the stimulation of MPS with [43] or without [44] increased protein intake, more specific measures and analyses of resistance training and physical activity could further our understanding of the effects of protein supplementation on muscle preservation during weight loss.

### 4.4. Effectiveness of Short-Term ADF on Blood Health Indices

The lack of a significant pre–post difference in blood pressure and fasting blood glucose suggests that short-term ADF does not benefit cardiovascular and diabetic health. Corroboratively, previous studies demonstrated that three weeks of ADF did not alter blood pressure [15], and two weeks of ADF did not improve fasting blood glucose levels [16]. While it is possible that the short-term ADF was simply not long enough to improve cardiovascular and diabetic health, longer-term (8–48 weeks) ADF studies have also reported inconsistent findings in this regard [10,11]. The lack of significant changes in these indices may also be due to the fact that our participants were young and that their average baseline SBP, DBP, and FG values were already within healthy ranges, limiting the potential for changes post-intervention. In addition, though transient, the significant reduction in DBP at the end of week three may be clinically important if the ADF protocol was sustained over a longer duration. Nonetheless, other biomarkers of cardiovascular and diabetic health, including plasma triglycerides, adiponectin levels, and insulin sensitivity, were not assessed in this study. Future studies should explore these variables to further our understanding of the effects of ADF on cardiovascular and diabetic health.

### 4.5. Limitations

The main limitation of this study is that the insufficient protein dose limited conclusions about the effectiveness of the protein supplementation strategy. Another limitation is that only young men were included; thus, the findings may not be generalizable to women or the older populations. Actual total weekly protein intake could not be determined, and personal preferences in dietary habits could have confounded the effects of protein supplementation, since food intake on feeding days were not controlled or monitored. Future studies could implement a controlled diet to ensure that the total protein intake across the duration of the study is matched between groups. It is also possible that hydration/dehydration statuses could have affected our BIA-derived body composition results [27]; nonetheless, our analyses of ICW/ECW indicated consistent and stable hydration statuses across time. Since GPAQ does not distinguish activity type (i.e., resistance vs. aerobic exercise), more specific measures and analyses of resistance training and physical activity could further elucidate our understanding of the effects of protein supplementation on muscle preservation during weight loss.

## 5. Conclusions

Short-term ADF is effective in reducing body mass and fat mass in normal weight to obese young men. However, this is accompanied by significant muscle loss, which cannot be attenuated by low-dose protein supplementation (25 g) during fasting days. Future studies should investigate the effectiveness of other interventions, such as protein or leucine supplementation throughout the ADF with controlled diets on both feeding and fasting days combined with controlled resistance-training protocols, to preserve or improve muscular outcomes during weight loss.

## Figures and Tables

**Table 1:** Participant characteristics for Study 1—single-arm group (S), Study 2—control (C), and protein (P) groups, and combined characteristics (All).

Characteristics		S (N = 13)		C (N = 13)		P (N = 11)		All (N = 37)	
		Mean	SD	Mean	SD	Mean	SD	Mean	SD
**Age (y)**	**Pre**	26	4	24	2	26	1	25	3
	**Post**	26	4	24	2	26	1	25	3
**Height (m)**	**Pre**	1.72	0.07	1.72	0.04	1.78	0.07	1.74	0.07
	**Post**	1.72	0.07	1.72	0.03	1.78	0.07	1.74	0.06
**Body Mass (kg)**	**Pre**	80.1	12.8	74.5	7.8	89.4	18.8	80.9	14.5
	**Post**	78.2	12.5	72.2	8.2	86.4	17.6	78.5	14.0
**Fat Mass (kg)**	**Pre**	19.8	7.6	16.4	5.9	23.4	12.2	19.7	9.0
	**Post**	18.8	7.6	14.6	6.2	21.3	12.0	18.1	8.9
**FFM (kg)**	**Pre**	60.3	7.2	58.1	4.8	66.0	8.7	61.2	7.5
	**Post**	59.4	6.7	57.6	5.2	65.1	8.0	60.5	7.2
**SMM (kg)**	**Pre**	34.2	4.2	32.8	2.9	37.4	5.1	34.6	4.4
	**Post**	33.6	4.0	32.6	3.2	36.8	4.7	34.2	4.3
**AFFM (kg)**	**Pre**	24.9	3.2	24.1	2.2	28.1	4.5	25.6	3.7
	**Post**	24.7	3.0	23.9	2.3	27.7	4.2	25.3	3.5
**FFMI (kg/m^2^)**	**Pre**	20.4	1.8	19.6	1.3	20.7	1.9	20.2	1.7
	**Post**	20.0	1.5	19.4	1.5	20.5	1.7	20.0	1.6
**SMMI (kg/m^2^)**	**Pre**	11.5	1.0	11.1	0.8	11.7	1.1	11.4	1.0
	**Post**	11.3	0.9	11.0	0.9	11.6	1.0	11.3	1.0
**AFFMI (kg/m^2^)**	**Pre**	8.4	0.7	8.1	0.5	8.8	0.9	8.4	0.7
	**Post**	8.3	0.5	8.1	0.6	8.7	0.8	8.3	0.7
**BMI (kg/m^2^)**	**Pre**	26.9	2.3	25.2	2.5	28.1	5.2	26.7	3.6
	**Post**	26.2	2.2	24.4	2.6	27.1	4.9	25.9	3.5
**PBF (%)**	**Pre**	24.2	5.8	21.7	6.0	25.1	7.7	23.6	6.4
	**Post**	23.4	5.8	19.8	6.6	23.6	8.0	22.2	6.8
**FMI (kg/m^2^)**	**Pre**	6.6	2.0	5.5	2.0	7.4	3.7	6.4	2.6
	**Post**	6.2	2.0	4.9	2.1	6.7	3.6	5.9	2.6
**VFA (cm^2^)**	**Pre**	81.2	33.3	67.7	25.4	96.8	44.3	81.1	35.6
	**Post**	76.1	33.2	59.1	26.7	88.3	44.2	73.8	35.9
**ICW/ECW**	**Pre**	1.7	0.0	1.7	0.0	1.7	0.0	1.7	0.0
	**Post**	1.7	0.0	1.7	0.0	1.7	0.0	1.7	0.0

Data are presented as means and standard deviations (SDs). FFM, fat-free mass; SMM, skeletal muscle mass; AFFM, appendicular fat-free mass; FFMI, fat-free mass index; SMMI, skeletal muscle mass index; AFFMI, appendicular fat-free mass index; BMI, body mass index; PBF, percentage body fat; FMI, fat mass index; VFA, visceral fat area; ICW, intracellular water; ECW, extracellular water.

**Table 2:** **Within**-subjects body composition analyses (combined data).

				SE	*p*-Value	Location	95% CI		Effect
Characteristics	Test	Statistic	df	Difference		Parameter ^a^	Lower	Upper	Size ^b^
**Body mass (kg)**	T-test	−9.378	36	0.252	<0.001	−2.4	−2.9	−1.9	−1.5
**Fat Mass (kg)**	T-test	−7.752	36	0.208	<0.001	−1.6	−2.0	−1.2	−1.3
**FFM (kg)**	T-test	−3.622	36	0.207	<0.001	−0.8	−1.2	−0.3	−0.6
**SMM (kg)**	T-test	−3.691	36	0.116	<0.001	−0.4	−0.7	−0.2	−0.6
**AFFM (kg)**	T-test	−2.842	36	0.088	0.007	−0.3	−0.4	−0.1	−0.5
**FFMI (kg/m^2^)**	Wilcoxon	117	36	0.068	<0.001	−0.2	−0.3	−0.1	−0.6
**SMMI (kg/m^2^)**	Wilcoxon	114	36	0.038	<0.001	−0.1	−0.2	−0.1	−0.7
**AFFMI (kg/m^2^)**	Wilcoxon	173	36	0.029	0.006	−0.1	−0.1	0.0	−0.5
**BMI (kg/m^2^)**	T-test	−9.603	36	0.083	<0.001	−0.8	−1.0	−0.6	−1.6
**FMI (kg/m^2^)**	T-test	−7.657	36	0.070	<0.001	−0.5	−0.7	−0.4	−1.3
**PBF (%)**	T-test	−5.531	36	0.247	<0.001	−1.4	−1.9	−0.9	−0.9
**VFA (cm^2^)**	T-test	−8.752	36	0.834	<0.001	−7.3	−9.0	−5.6	−1.4

^a^ indicates a mean difference for the *t*-test or a Hodges–Lehmann median difference for Wilcoxon. ^b^ indicates Cohen’s d for *t*-test or a matched rank biserial correlation for Wilcoxon. FFM, fat-free mass; SMM, skeletal muscle mass; AFFM, appendicular fat-free mass; FFMI, fat-free mass index; SMMI, skeletal muscle mass index; AFFMI, appendicular fat-free mass index; BMI, body mass index; PBF, percentage body fat; FMI, fat mass index; VFA, visceral fat area.

**Table 3:** Weekly physical activity and health indices (combined data).

	Week	N	Mean	SD
**GPAQ (MET·min/week)**	**0**	37	1443	1053
	**1**	37	1336	1051
	**2**	37	1089	823
	**3**	37	1139	800
	**4**	37	1189	1012
**SBP (mmHg)**	**0**	37	118	10
	**1**	37	118	9
	**2**	37	117	8
	**3**	37	116	8
	**4**	37	117	10
**DBP (mgHg)**	**0**	37	69	9
	**1**	37	68	8
	**2**	37	67	8
	**3**	37	67	9
	**4**	37	68	8
**FG (mmol)**	**0**	36	5.3	0.4
	**1**	37	5.1	0.3
	**2**	37	5.3	0.4
	**3**	36	5.1	0.4
	**4**	37	5.2	0.4

Data are presented as means and standard deviations (SDs). GPAQ, global physical activity questionnaire; MET, metabolic equivalent of task; SBP, systolic blood pressure; DBP, diastolic blood pressure; FG, fasting blood glucose.

## Data Availability

The raw data supporting the conclusions of this article will be made available by the authors on request.

## Abbreviations

The following abbreviations are used in this manuscript:

ADF	Alternate-day fasting
AFFM	Appendicular fat-free mass
AFFMI	Appendicular fat-free mass index
BIA	Bioelectrical impedance analysis
BMI	Body mass index
BP	Blood pressure
C	Control group
CVD	Cardiovascular disease
DBP	Diastolic blood pressure
FFM	Fat-free mass
FFMI	Fat-free mass index
FG	Fasting blood glucose
FM	Fat mass
FMI	Fat mass index
GEE	Generalized estimating equation
GPAQ	Global physical activity questionnaire
ICW/ECW	Intracellular water/extracellular water
IF	Intermittent fasting
IRB	Institutional review board
MET	Metabolic equivalent of task
MPS	Muscle protein synthesis
NTU	Nanyang technological university
P	Protein group
PBF	Percentage of body fat
rANOVA	Repeated-measures analysis of variance (ANOVA)
RCT	Randomized, controlled trial
S	Single-arm study
SBP	Systolic blood pressure
SMM	Skeletal muscle mass
SMMI	Skeletal muscle mass index
VFA	Visceral fat area
VLCD	Very-low-calorie dieting

## References

[1] WHO Global Health Observatory (GHO) Data—Overweight and Obesity 2022. https://www.who.int/gho/ncd/risk_factors/overweight_obesity/obesity_adults/en/.

[2] WHO (2004). Appropriate body-mass index for Asian populations and its implications for policy and intervention strategies. Lancet.

[3] Chen K.K., Wee S.-L., Pang B., Lau L.K., Jabbar K.A., Seah W.T., Ng T.P. (2021). Relationship between BMI with percentage body fat and obesity in Singaporean adults—The Yishun Study. BMC Public Health.

[4] Wang Y.C., McPherson K., Marsh T., Gortmaker S.L., Brown M. (2011). Health and economic burden of the projected obesity trends in the USA and the UK. Lancet.

[5] Meng H., Zhu L., Kord-Varkaneh H., Santos H.O., Tinsley G.M., Fu P. (2020). Effects of intermittent fasting and energy-restricted diets on lipid profile: A systematic review and meta-analysis. Nutrition.

[6] Cho Y., Hong N., Kim K.W., Cho S.J., Lee M., Lee Y.H., Lee Y.H., Kang E.S., Cha B.S., Lee B.W. (2019). The Effectiveness of Intermittent Fasting to Reduce Body Mass Index and Glucose Metabolism: A Systematic Review and Meta-Analysis. J. Clin. Med..

[7] Welton S., Minty R., O’Driscoll T., Willms H., Poirier D., Madden S., Kelly L. (2020). Intermittent fasting and weight loss: Systematic review. Can. Fam. Physician.

[8] Patterson R.E., Sears D.D. (2017). Metabolic Effects of Intermittent Fasting. Annu. Rev. Nutr..

[9] Varady K.A., Bhutani S., Klempel M.C., Kroeger C.M., Trepanowski J.F., Haus J.M., Hoddy K.K., Calvo Y. (2013). Alternate day fasting for weight loss in normal weight and overweight subjects: A randomized controlled trial. Nutr. J..

[10] Park J., Seo Y.G., Paek Y.J., Song H.J., Park K.H., Noh H.M. (2020). Effect of alternate-day fasting on obesity and cardiometabolic risk: A systematic review and meta-analysis. Metab. Clin. Exp..

[11] Cui Y., Cai T., Zhou Z., Mu Y., Lu Y., Gao Z., Wu J., Zhang Y. (2020). Health Effects of Alternate-Day Fasting in Adults: A Systematic Review and Meta-Analysis. Front. Nutr..

[12] Alhamdan B.A., Garcia-Alvarez A., Alzahrnai A.H., Karanxha J., Stretchberry D.R., Contrera K.J., Utria A.F., Cheskin L.J. (2016). Alternate-day versus daily energy restriction diets: Which is more effective for weight loss? A systematic review and meta-analysis. Obes. Sci. Pract..

[13] Trepanowski J.F., Kroeger C.M., Barnosky A., Klempel M.C., Bhutani S., Hoddy K.K., Gabel K., Freels S., Rigdon J., Rood J. (2017). Effect of Alternate-Day Fasting on Weight Loss, Weight Maintenance, and Cardioprotection Among Metabolically Healthy Obese Adults: A Randomized Clinical Trial. JAMA Intern. Med..

[14] Stekovic S., Hofer S.J., Tripolt N., Aon M.A., Royer P., Pein L., Stadler J.T., Pendl T., Prietl B., Url J. (2019). Alternate Day Fasting Improves Physiological and Molecular Markers of Aging in Healthy, Non-obese Humans. Cell Metab..

[15] Heilbronn L.K., Smith S.R., Martin C.K., Anton S.D., Ravussin E. (2005). Alternate-day fasting in nonobese subjects: Effects on body weight, body composition, and energy metabolism. Am. J. Clin. Nutr..

[16] Halberg N., Henriksen M., Soderhamn N., Stallknecht B., Ploug T., Schjerling P., Dela F. (2005). Effect of intermittent fasting and refeeding on insulin action in healthy men. J. Appl. Physiol..

[17] Klempel M.C., Bhutani S., Fitzgibbon M., Freels S., Varady K.A. (2010). Dietary and physical activity adaptations to alternate day modified fasting: Implications for optimal weight loss. Nutr. J..

[18] Templeman I., Smith H.A., Chowdhury E., Chen Y.C., Carroll H., Johnson-Bonson D., Hengist A., Smith R., Creighton J., Clayton D. (2021). A randomized controlled trial to isolate the effects of fasting and energy restriction on weight loss and metabolic health in lean adults. Sci. Transl. Med..

[19] Varady K.A., Bhutani S., Church E.C., Klempel M.C. (2009). Short-term modified alternate-day fasting: A novel dietary strategy for weight loss and cardioprotection in obese adults. Am. J. Clin. Nutr..

[20] Derron N., Güntner A.T., Weber I.C., Braun J., Koska İ.Ö., Othman A., Mönch L., von Eckardstein A., Puhan M.A., Beuschlein F. (2025). Alternate-day fasting elicits larger changes in fat mass than time-restricted eating in adults without obesity—A randomized clinical trial. Clin. Nutr..

[21] Aragon A.A., Schoenfeld B.J., Wildman R., Kleiner S., VanDusseldorp T., Taylor L., Earnest C.P., Arciero P.J., Wilborn C., Kalman D.S. (2017). International society of sports nutrition position stand: Diets and body composition. J. Int. Soc. Sports Nutr..

[22] Westerterp-Plantenga M.S., Lemmens S.G., Westerterp K.R. (2012). Dietary protein—Its role in satiety, energetics, weight loss and health. Br. J. Nutr..

[23] Wycherley T.P., Moran L.J., Clifton P.M., Noakes M., Brinkworth G.D. (2012). Effects of energy-restricted high-protein, low-fat compared with standard-protein, low-fat diets: A meta-analysis of randomized controlled trials. Am. J. Clin. Nutr..

[24] Soenen S., Bonomi A.G., Lemmens S.G., Scholte J., Thijssen M.A., van Berkum F., Westerterp-Plantenga M.S. (2012). Relatively high-protein or ‘low-carb’ energy-restricted diets for body weight loss and body weight maintenance?. Physiol. Behav..

[25] Backx E.M.P., Tieland M., Borgonjen-van den Berg K.J., Claessen P.R., van Loon L.J.C., de Groot L.C.P.G.M. (2016). Protein intake and lean body mass preservation during energy intake restriction in overweight older adults. Int. J. Obes..

[26] Bhutani S., Klempel M.C., Kroeger C.M., Trepanowski J.F., Varady K.A. (2013). Alternate day fasting and endurance exercise combine to reduce body weight and favorably alter plasma lipids in obese humans. Obesity.

[27] Faria S.L., Faria O.P., Cardeal M.D., Ito M.K. (2014). Validation study of multi-frequency bioelectrical impedance with dual-energy X-ray absorptiometry among obese patients. Obes. Surg..

[28] Ramírez-Vélez R., Tordecilla-Sanders A., Correa-Bautista J.E., González-Ruíz K., González-Jiménez E., Triana-Reina H.R., García-Hermoso A., Schmidt-RioValle J. (2018). Validation of multi-frequency bioelectrical impedance analysis versus dual-energy X-ray absorptiometry to measure body fat percentage in overweight/obese Colombian adults. Am. J. Hum. Biol..

[29] Bosy-Westphal A., Later W., Hitze B., Sato T., Kossel E., Gluer C.C., Heller M., Muller M.J. (2008). Accuracy of bioelectrical impedance consumer devices for measurement of body composition in comparison to whole body magnetic resonance imaging and dual X-ray absorptiometry. Obes. Facts.

[30] Bhutani S., Klempel M.C., Berger R.A., Varady K.A. (2010). Improvements in coronary heart disease risk indicators by alternate-day fasting involve adipose tissue modulations. Obesity.

[31] Armstrong T., Bull F. (2006). Development of the World Health Organization Global Physical Activity Questionnaire (GPAQ). J. Public Health.

[32] NHLBI Obesity Education Initiative Expert Panel on the Identification, Evaluation, and Treatment of Obesity in Adults (US). Clinical Guidelines on the Identification, Evaluation, and Treatment of Overweight and Obesity in Adults: The Evidence Report. https://www.ncbi.nlm.nih.gov/books/NBK2003/.

[33] Volek J.S., Vanheest J.L., Forsythe C.E. (2005). Diet and exercise for weight loss: A review of current issues. Sports Med..

[34] Dohm G.L., Beeker R.T., Israel R.G., Tapscott E.B. (1986). Metabolic responses to exercise after fasting. J. Appl. Physiol..

[35] Kreitzman S.N., Coxon A.Y., Szaz K.F. (1992). Glycogen storage: Illusions of easy weight loss, excessive weight regain, and distortions in estimates of body composition. Am. J. Clin. Nutr..

[36] Davis C.S., Clarke R.E., Coulter S.N., Rounsefell K.N., Walker R.E., Rauch C.E., Huggins C.E., Ryan L. (2016). Intermittent energy restriction and weight loss: A systematic review. Eur. J. Clin. Nutr..

[37] Cava E., Yeat N.C., Mittendorfer B. (2017). Preserving Healthy Muscle during Weight Loss. Adv. Nutr..

[38] Larsen A.E., Bibby B.M., Hansen M. (2018). Effect of a Whey Protein Supplement on Preservation of Fat Free Mass in Overweight and Obese Individuals on an Energy Restricted Very Low Caloric Diet. Nutrients.

[39] Wolfe R.R., Miller S.L. (2008). The Recommended Dietary Allowance of Protein: A Misunderstood Concept. JAMA.

[40] Churchward-Venne T.A., Breen L., Di Donato D.M., Hector A.J., Mitchell C.J., Moore D.R., Stellingwerff T., Breuille D., Offord E.A., Baker S.K. (2014). Leucine supplementation of a low-protein mixed macronutrient beverage enhances myofibrillar protein synthesis in young men: A double-blind, randomized trial. Am. J. Clin. Nutr..

[41] Devries M.C., McGlory C., Bolster D.R., Kamil A., Rahn M., Harkness L., Baker S.K., Phillips S.M. (2018). Leucine, Not Total Protein, Content of a Supplement Is the Primary Determinant of Muscle Protein Anabolic Responses in Healthy Older Women. J. Nutr..

[42] Weigle D.S., Breen P.A., Matthys C.C., Callahan H.S., Meeuws K.E., Burden V.R., Purnell J.Q. (2005). A high-protein diet induces sustained reductions in appetite, ad libitum caloric intake, and body weight despite compensatory changes in diurnal plasma leptin and ghrelin concentrations. Am. J. Clin. Nutr..

[43] Yang Y., Breen L., Burd N.A., Hector A.J., Churchward-Venne T.A., Josse A.R., Tarnopolsky M.A., Phillips S.M. (2012). Resistance exercise enhances myofibrillar protein synthesis with graded intakes of whey protein in older men. Br. J. Nutr..

[44] Frimel T.N., Sinacore D.R., Villareal D.T. (2008). Exercise attenuates the weight-loss-induced reduction in muscle mass in frail obese older adults. Med. Sci. Sports Exerc..

